# Predictors of Colorectal Cancer Screening in Two Underserved U.S. Populations: A Parallel Analysis

**DOI:** 10.3389/fonc.2018.00230

**Published:** 2018-06-19

**Authors:** Brittany M. Bernardo, Amy L. Gross, Gregory Young, Ryan Baltic, Sarah Reisinger, William J. Blot, Electra Diane Paskett

**Affiliations:** ^1^Comprehensive Cancer Center, The Ohio State University, Columbus, OH, United States; ^2^Vanderbilt Institute for Clinical and Translational Research, International Epidemiology Field Station, Vanderbilt University Medical Center, Nashville, TN, United States; ^3^Center for Biostatistics, The Ohio State University, Columbus, OH, United States; ^4^Department of Medicine, Division of Epidemiology, School of Medicine, Vanderbilt University, Nashville, TN, United States; ^5^Division of Cancer Prevention and Control, Department of Internal Medicine, College of Medicine, The Ohio State University, Columbus, OH, United States; ^6^Division of Epidemiology, College of Public Health, The Ohio State University, Columbus, OH, United States

**Keywords:** guideline colorectal cancer screening, underserved populations, neighborhood deprivation, guideline screening, correlates of screening

## Abstract

**Background:**

Despite declining colorectal cancer (CRC) incidence and mortality rates in the U.S., significant geographic and racial disparities in CRC death rates remain. Differences in guideline-concordant CRC screening rates may explain some of these disparities. We aim to assess individual and neighborhood-level predictors of guideline-concordant CRC screening within two cohorts of individuals located within CRC mortality geographic hotspot regions in the U.S.

**Methods:**

A total of 36,901 participants from the Southern Community Cohort Study and 4,491 participants from the Ohio Appalachia CRC screening study were included in this study. Self-reported date of last CRC screening was used to determine if the participant was within guidelines for screening. Logistic regression models were utilized to determine the association of individual-level predictors, neighborhood deprivation, and residence in hotspot regions on the odds of being within guidelines for CRC screening.

**Results:**

Lower household income, lack of health insurance, and being a smoker were each associated with lower odds of being within guidelines for CRC screening in both cohorts. Area-level associations were less evident, although up to 15% lower guideline adherence was associated with residence in neighborhoods of greater deprivation and in the Lower Mississippi Delta, one of the identified CRC mortality hotspots.

**Conclusion:**

These results reveal the adverse effects of lower area-level and individual socioeconomic status on adherence to CRC guideline screening.

## Introduction

Colorectal cancer (CRC) is the second most common type of cancer and cause of cancer death in the U.S., combining cases for men and women together (1). While mortality rates have declined in recent years, predominantly due to increases in screening and improvements in treatment, this cancer is still responsible for a large portion of cancer incidence, mortality, cost, and pain and suffering (2, 3). Some regions of the U.S. suffer from higher CRC incidence and mortality than others. Specifically, Siegel et al. (4) used spatial mapping techniques to identify three hotspot regions in the U.S. with increased CRC mortality—the Lower Mississippi Delta, West Central Appalachia, and Eastern Virginia/North Carolina. From 2009 to 2011, CRC death rates in these hotspot regions were between 9 and 40% higher than non-hotspot regions of the U.S. Reasons for elevated CRC death rates in these regions could include lower CRC screening rates, poor access to treatment, lower socioeconomic status (SES), as well as increased obesity and poor diet quality (5). These hotspot regions have several factors in common: (1) as they all include areas of widespread poverty (6–8), (2) are mainly rural and/or Appalachian (9), (3) have pockets of minority residents, and (4) are in health-care professional shortage areas (10, 11).

To assess the impact of individual and neighborhood-level factors on CRC screening, we used data from two NCI-funded studies, the Southern Community Cohort Study (SCCS) [R01 CA092447] and Community-based Participatory Research Strategies to Increase Colorectal Cancer Screening in Ohio Appalachia (hereafter referred to as the Ohio Appalachia study) [R24MD002785]. These studies included portions of the three hotspot areas—notably the Mississippi Delta and West Central Appalachia—and involved assessment of CRC screening rates and predictors of being within guidelines for CRC screening.

## Materials and Methods

### Ohio Appalachia (OA) Study Population

Twelve counties in Appalachian Ohio were selected based on a higher than average incidence of late-stage CRC. A list of residents in each county was provided by InfoUSA County Directories. All residents between 51 and 99 years of age were selected. More male than female residents were represented in the provided lists, so a proportional sampling scheme was used to randomly select names by county to represent the county gender proportions in the 2000 U.S. census. Names were sampled with replacement annually over a period of 4 years (2009–2013) during each of four study waves. Participants were eligible if they were 51–75 years of age; able to read and speak English; able to provide informed consent; a resident of 1 of the 12 counties selected in OA; had a working telephone number; no prior history of CRC, familial or hereditary cancer, polyps or irritable bowel disease; in good health; and not pregnant. Potential participants were mailed an informational packet and a letter indicating that someone would be calling them within the next week to conduct a short telephone survey. Informed consent was obtained from each participant, and participants received a $10 gift card after completing a telephone survey. From 2009 to 2013, a total of 23,297 letters were mailed to potential participants in four study waves. Of these individuals, 6,405 were determined to be ineligible, 6,012 were unable to be contacted, 703 were deceased, and 5,686 refused participation, leaving a total of 4,491 participants who consented to the study. More details regarding recruitment procedures can be found elsewhere (12, 13). This study was approved by the Ohio State University Institutional Review Board.

### SCCS Population

The SCCS is a prospective cohort study designed to identify factors underlying racial, geographic, and socioeconomic disparities in cancer and other health outcomes in a medically underserved population (14). Recruitment took place from 2002 to 2009 within 12 states in the southeastern U.S. The majority of participants (85%) were recruited and enrolled in person at community health centers, with the remaining 15% of participants recruited and enrolled through mailings to age-, sex-, and race-stratified random samples of the general population. Participants were eligible if they were between 40 and 79 years old, English-speaking, and not undergoing treatment for any cancer (excluding non-melanoma skin cancer) within the past 12 months.

At enrollment, participants completed a baseline questionnaire with a trained interviewer to assess demographics, insurance coverage status/type, personal and family medical history, lifestyle behaviors, and cancer screening history. Participants enrolling through the mail completed an identical survey on a scannable form. The SCCS was approved by Vanderbilt University Medical Center and Meharry Medical College Institutional Review Boards.

While over 84,000 participants enrolled in the SCCS, due to our focus on CRC screening, we excluded participants who were under age 50 years at enrollment (*n* = 37,326), those with a history of CRC (*n* = 425), polyps (*n* = 4,761), or irritable bowel disease (*n* = 278), those with missing information on colonoscopy or sigmoidoscopy screening (*n* = 633) or race (*n* = 219), and those with a family history of CRC in at least one first-degree relative (*n* = 2,858). Those with a family history are excluded in both cohorts because they may have an increased risk for CRC, and therefore should adhere to a different screening regimen than the general population. The final study population for this analysis consisted of 36,901 participants.

### CRC Screening Within Guidelines

To be consistent with the United States Preventive Service Task Force guidelines (15), participants were considered as within guidelines for CRC screening if they reported completing either (1) a flexible sigmoidoscopy in the past 5 years or (2) a colonoscopy in the past 10 years.

### Individual-Level Characteristics

Participants self-reported characteristics such as race, income, educational attainment, smoking status, and insurance status. Due to lack of variability in race in the OA population, race was categorized as white or other. SCCS participants self-identified race with the option to select more than one category. For analytic purposes, race was categorized as black, white, or other, which included individuals self-identifying as both black and white. Participants self-reported annual household income which was categorized as “low,” “middle,” and “high.” For OA income, categories were <$20,000, $20,000–$50,000, and >$50,000, respectively, while SCCS categories were <$15,000, $15,000–$49,999, and >$50,000, respectively. Individuals who reported smoking 100 cigarettes in their lifetime but not currently smoking were categorized as “former” smokers, individuals who did not currently smoke and smoked less than 100 cigarettes in their lifetime were categorized as “never” smokers, and individuals who currently smoked cigarettes were classified as “current” smokers.

### Neighborhood Deprivation Index (NDI)

For both cohorts, neighborhood SES was estimated using a NDI with methods described elsewhere (16). Briefly, the index was based on 20 variables at the census tract-level (for SCCS) or county-level variables (OA) related to poverty, housing, occupation, employment, and education. SCCS participant addresses at baseline were geocoded and linked to the tract-level variables from the 2000 U.S. census. Principal components analysis was used to determine which census variables would be retained due to high factor loadings. The resulting 11 census variables were included in the final principal components analysis to generate the NDI, a continuous measure, with higher values representing more deprivation. The same 11 components were used in the calculation of the NDI for the OA study. In order to maintain consistency with the time period in which the data were collected, the American Community Survey 5-year estimates for 2009–2013 were used instead of the 2000 U.S. census.

### Hotspot/Geographic Regions

Using data on CRC mortality “hotspots” as defined by Siegel et al. (4), we categorized all counties as belonging to “non-hotspot” versus “any hotspot,” as well as Hotspot 1 (Lower Mississippi Delta), Hotspot 2 (West Central Appalachia), or Hotspot 3 (Eastern Virginia/North Carolina). In addition, data from the Appalachian Regional Commission (17) were used to designate counties as Appalachian versus non-Appalachian.

### Statistical Analysis

Standard and mixed-effects logistic regression models were used to evaluate the outcome of self-reported CRC screening within guidelines for flexible sigmoidoscopy or colonoscopy. Individual-level predictors (e.g., age, education) and NDI were evaluated separately in univariable models. For the NDI, a mixed-effects logistic regression approach was used with a random effect for county (OA) and census tract (SCCS). For the OA study, a multivariable model was built by using a backwards selection process on the individual-level predictors (requiring a *p*-value of less than 0.05 for retention), then adding the NDI. For SCCS, covariates adjusted for in the multivariable model included age at enrollment, sex, race, education, employment, smoking status, insurance status, income, deprivation index, and hotspot.

To determine the impact of age on screening within guidelines, separate logistic regression models were fit stratified by age <65 compared with participants age 65 or older. The main analyses used CRC screening within guidelines as the outcome; however, sigmoidoscopy within guidelines and colonoscopy within guidelines were examined individually as alternative outcomes for the SCCS study. Analyses were conducted in SAS v9.3, 9.4 (SAS Institute, Cary, NC, USA) and Stata (version 14; StataCorp LP, College Station, TX, USA).

## Results

### OA Participant Characteristics

Participant characteristics are reported in Table 1. Out of 4,491 total participants, 2,482 (55.3%) reported having received CRC screening within guidelines. Among participants who received guideline screening, most (94.8%) received a colonoscopy. Most participants were white (95.9%), female (57.4%), had at least some college education (57.3%), and had health insurance (90.2%). A greater percentage (21.8%) of individuals not within guidelines were current smokers compared with participants within guidelines (9.1%).

**Table 1:** Characteristics of Ohio Appalachia (OA) and Southern Community Cohort Study (SCCS) participants by colorectal cancer guideline-concordant screening status.

Characteristic	All participants		Within guidelines		Outside of guidelines	
	OA (*n* = 4,491) *n*(%)	SCCS (*n* = 36,901) *n*(%)	OA (*n* = 2,482) *n*(%)	SCCS (*n* = 12,182) *n*(%)	OA (*n* = 2,009) *n*(%)	SCCS (*n* = 24,719) *n*(%)
Sex						
Female	2,579 (57.4)	22,060 (59.8)	1,419 (57.2)	7,541 (61.9)	1,160 (57.7)	14,519 (58.7)
Male	1,912 (42.6)	14,841 (40.2)	1,063 (42.8)	4,641 (38.1)	849 (42.3)	10,200 (41.3)

Age (mean, SD)	61.8 (6.7)	58.1 (6.7)	62.5 (6.6)	59.9 (7.0)	60.9 (6.7)	57.2 (6.4)

Race						
White	4,309 (95.9)	11,625 (31.5)	2,386 (96.1)	4,172 (34.3)	1,923 (95.7)	7,453 (30.2)
Black	^a^	23,832 (64.6)	^a^	7,520 (61.7)	^a^	16,312 (66.0)
Other	176 (3.9)	1,444 (3.9)	93 (3.7)	490 (4.0)	83 (4.1)	954 (3.9)

Education						
High school graduate	1,556 (34.6)	11,072 (30.0)	823 (33.2)	3,295 (27.1)	733 (36.5)	7,777 (31.5)
Some college or more	2,572 (57.3)	14,042 (38.1)	1,505 (60.6)	5,434 (44.6)	1,067 (53.1)	8,608 (34.8)
Less than high school	360 (8.0)	11,759 (31.9)	151 (6.1)	3,443 (28.3)	209 (10.4)	8,316 (33.6)
Missing	3 (0.1)	28 (0.1)	3 (0.1)	10 (0.1)	0 (0.0)	18 (0.1)

Employment status						
Unemployed/disabled	724 (16.1)	24,009 (65.1)	330 (13.3)	8,036 (66.0)	394 (19.6)	15,973 (64.6)
Retired/volunteer	1,826 (40.7)	–	1,118 (45.0)	–	708 (35.2)	–
Full/part time	1,932 (43.0)	12,757 (34.6)	1,031 (41.5)	4,091 (33.6)	901 (44.8)	8,666 (35.1)
Missing	9 (0.2)	135 (0.4)	3 (0.1)	55 (0.5)	6 (0.3)	80 (0.3)

Insurance status						
Public	1,717 (38.2)	13,106 (35.5)	1,039 (41.9)	4,927 (40.4)	678 (33.7)	8,179 (33.1)
Private	2,336 (52.0)	10,030 (27.2)	1,342 (54.1)	4,422 (36.3)	994 (49.5)	5,608 (22.7)
Uninsured	411 (9.2)	12,367 (33.5)	87 (3.5)	2,356 (19.4)	324 (16.1)	10,011 (40.5)
Other	–	1,232 (3.3)	–	426 (3.5)	–	806 (3.3)
Missing	27 (0.6)	166 (0.5)	14 (0.6)	51 (0.4)	13 (0.6)	115 (0.5)

Smoking status						
Former smoker	1,485 (33.1)	10,216 (27.7)	853 (34.4)	4,013 (32.9)	632 (31.5)	6,203 (25.1)
Current smoker	664 (14.8)	12,314 (33.4)	227 (9.1)	2,885 (23.7)	437 (21.8)	9,429 (38.1)
Never smoker	2,329 (51.9)	14,099 (38.2)	1,394 (56.2)	5,164 (42.4)	935 (46.5)	8,935 (36.2)
Missing	13 (0.3)	272 (0.7)	8 (0.3)	120 (1.0)	5 (0.2)	152 (0.6)

Income^b^						
Low	744 (16.6)	20,186 (54.7)	305 (12.3)	5,671 (46.6)	439 (21.9)	14,515 (58.7)
Middle	1,540 (34.3)	12,397 (33.6)	826 (33.3)	4,336 (35.6)	714 (35.5)	8,061 (32.6)
High	1,691 (37.7)	3,720 (10.1)	1,081 (43.6)	1,961 (16.1)	610 (30.4)	1,759 (7.1)
Missing	516 (11.5)	598 (1.6)	270 (10.9)	214 (1.8)	246 (12.2)	384 (1.6)

Geographic region						
Appalachia	4,491 (100)	6,200 (16.8)	2,482 (100)	2,210 (18.2)	2,009 (100)	3,990 (16.2)
Hotspot, any	2,579 (57.4)	11,361 (30.8)	1,414 (57.0)	3,603 (29.6)	1,165 (58.0)	7,758 (31.4)
Hotspot 1, Lower MS Delta	–	7,218 (19.6)	–	2,007 (16.5)	–	5,211 (21.1)
Hotspot 2, West Central Appalachia	–	3,753 (10.2)	–	1,419 (11.7)	–	2,334 (9.4)
Hotspot 3, NC/VA	–	390 (1.1)	–	177 (1.5)	–	213 (0.9)

*^a^OA race: due to lack of variability in race, it was categorized as white and other*.

*^b^Income ranges were not able to be harmonized, so each study categorized their results into low, middle, and high*.

### SCCS Participant Characteristics

Among the 36,901 SCCS participants included in this analysis, 33.0% (*n* = 12,182) reported having been screened for CRC within guidelines. Factors associated with increased likelihood of screening included female sex, older age, white race, higher educational attainment, insurance coverage, and higher household income (Table 1). The proportion of black participants who reported having received guideline-concordant screening was lower than what was reported by white participants (31.6 versus 35.9%, respectively; *p* < 0.001). Notably, of those participants who were screened within guidelines, 60.9% had received colonoscopy only, 18.1% had received flexible sigmoidoscopy only, and 20.2% had received both colonoscopy and flexible sigmoidoscopy. Nearly one-third of participants were living in a “hotspot” for CRC mortality at baseline.

### OA Correlates of Being Within Guidelines for CRC Screening

A county-level socioeconomic index (NDI) was included in univariable and multivariable models, but was not found to be significantly associated with obtaining guideline CRC screening in either model (*p* = 0.60 and *p* = 0.66, respectively). After stratifying our logistic regression models by age <65 compared with those 65 or older, it was determined that these stratified results were similar to those from the pooled analyses. Therefore, we present only the pooled results here.

Following backwards selection, older age, public or private insurance, and higher income were associated with higher odds of being within screening guidelines. Current smokers were significantly less likely to receive guideline screening than never smokers in the adjusted models. Out of the 12 counties in the OA study, 7 counties were considered as “hotspot” areas for CRC incidence and mortality. In a multivariable model, an indicator variable for residence in a hotspot county was not found to be significantly associated with receipt of guideline-concordant CRC screening [OR (95% CI): 0.97 (0.75, 1.24); *p* = 0.79].

### SCCS Correlates of Being Within Guidelines for CRC Screening

Similar to the OA findings, older age, higher education, higher income and having health insurance were associated with adherence to CRC screening guidelines within the SCCS cohort (Table 2). In contrast to Ohio, women in the SCCS were more likely to be within guidelines. Similar to the OA study, age-stratified results were similar to pooled results. Thus, only pooled results are presented here. After adjusting for age, sex, insurance status, education, income, employment status, smoking, and deprivation index, black race was associated with significantly increased odds of screening within guidelines [OR: 1.22, 95% CI: (1.15–1.30); *p* < 0.001]. In the same model, residence in the Lower Mississippi Delta hotspot was associated with decreased likelihood of screening [OR: 0.85, 95% CI: (0.79–0.91); *p* < 0.001], but residence in either of the other two CRC mortality hotspots was associated with increased likelihood of screening, compared with the reference group of non-hotspot [OR: 1.35, 95% CI: (1.24–1.47), *p* < 0.001; OR: 1.70, 95% CI: (1.36–2.12), *p* < 0.001]. Increasing neighborhood deprivation was significantly associated with reduced odds of guideline-concordant screening [OR: 0.93, 95% CI: (0.91–0.95); *p* < 0.001] per unit NDI increase.

**Table 2:** Multivariable logistic regression results for Ohio Appalachia (OA) and Southern Community Cohort Study (SCCS) participants’ receipt of colorectal cancer screening within guidelines.

	Multivariable OR (95% CI)	

Characteristic	OA *n* = 3,934	SCCS *n* = 36,345
Sex		
Male	–	1.00
Female	–	1.10 (1.04, 1.15)

Age	1.02 (1.01, 1.04)	1.05 (1.04, 1.05)

Race		
White	–	1.00
Other	–	1.00 (0.88, 1.14)
Black		1.22 (1.15, 1.30)

Education		
Less than high school	–	1.00
High school graduate	–	1.08 (1.01, 1.15)
Some college or more		1.31 (1.22, 1.39)

Employment status		
Unemployed/disabled	1.00	1.00
Retired/volunteer	1.08 (0.87, 1.35)	–
Full/part time	0.85 (0.68, 1.05)	0.77 (0.72, 0.81)

Insurance status		
Uninsured	1.00	1.00
Public	3.47 (2.57, 4.68)	1.96 (1.83, 2.08)
Private	3.27 (2.48, 4.32)	2.16 (2.01, 2.33)

Smoking status		
Never smoker	1.00	1.00
Former smoker	0.90 (0.78, 1.04)	1.08 (1.02, 1.14)
Current smoker	0.44 (0.36, 0.53)	0.73 (0.69, 0.78)

Income		
Low	1.00	1.00
Middle	1.43 (1.17, 1.75)	1.21 (1.14, 1.29)
High	2.26 (1.81, 2.83)	1.97 (1.79, 2.17)

Deprivation index	–	0.93 (0.91, 0.95)

Hotspot	–	
Non-hotspot		1.00
Lower MS Delta		0.85 (0.79, 0.91)
West Central Appalachia		1.35 (1.24, 1.47)
East NC/VA		1.70 (1.36, 2.12)

Given the greater proportion of blacks self-reporting receipt of flexible sigmoidoscopy-only compared with whites (21.6 versus 12.3%; *p* < 0.001), we conducted an analysis examining the association between race and hotspot with the outcome of colonoscopy within guidelines (Table 3). In adjusted multivariable multi-level models, we found that the association between black race and screening outcome was substantially attenuated [OR: 1.07, 95% CI: (1.01–1.15); *p* = 0.03], while the associations for NDI and hotspot regions remained similar to what was seen in Table 2.

**Table 3:** Southern Community Cohort Study multivariable multi-level logistic regression results for colonoscopy-only screening within guidelines.

Characteristic	Multivariable OR (95% CI)
Sex	
Male	1.00
Female	1.19 (1.13, 1.26)

Age	1.04 (1.04, 1.05)

Race	
White	1.00
Black	1.07 (1.01, 1.15)
Other	1.03 (0.90, 1.18)

Education	
Less than high school	1.00
High school graduate	1.14 (1.07, 1.22)
Some college or more	1.35 (1.26, 1.44)

Employment status	
Unemployed	1.00
Currently employed	0.74 (0.70, 0.80)

Insurance status	
Uninsured	1.00
Public	2.04 (1.91, 2.19)
Private	2.33 (2.15, 2.52)

Smoking status	
Never smoker	1.00
Former smoker	1.07 (1.01, 1.14)
Current smoker	0.74 (0.70, 0.80)

Income	
<$15,000	1.00
$15,000–$50,000	1.23 (1.15, 1.31)
>$50,000	1.88 (1.70, 2.08)
Deprivation index	0.92 (0.89, 0.94)

Hotspot	
Non-hotspot	1.00
Lower MS Delta	0.85 (0.78, 0.93)
West Central Appalachia	1.48 (1.36, 1.62)
East NC/VA	1.63 (1.29, 2.06)

## Discussion

The goal of this study was to determine predictors of being within guidelines for CRC screening, using data from two populations located within hotspot regions for CRC mortality. The proportion of participants receiving within-guideline CRC screening was 55% in the OA study (assessed from 2009 to 2013) and 33% in SCCS (assessed from 2002 to 2009), both of which are lower than the 59% of U.S. adults who received either FOBT or endoscopy as reported by the National Health Interview Survey in 2010 (18). Some of the difference in screening rates could be due to secular trends, being that SCCS started recruitment in 2002 while the OA study began in 2009. One potential explanation for such low rates of guideline CRC screenings in these two populations is that we did not include receipt of guideline FOBT/FIT screenings in our models, which may artificially lower the percentage of individuals within guidelines for screening. However, this is not the case in the OA study, as only 4.2% of participants who were within guidelines for CRC screening by medical record review were within guidelines solely by FOBT/FIT.

In both studies, we found that lower household income, lack of health insurance, and current smoking were significantly and independently associated with reduced likelihood of screening. Within SCCS, area-level measures of deprivation were also significantly associated with receipt of guideline CRC screening, with participants residing in census tracts of higher socioeconomic deprivation (as measured by the NDI index) significantly less likely to be within guidelines for CRC screening. We hypothesize that the NDI was not significantly associated with guideline adherence in the OA population due to the smaller sample size of 12 Ohio counties. Interestingly, while black race was associated with reduced likelihood of screening in unadjusted models, the reverse association was seen in fully adjusted multi-level models, with blacks having 1.20 (95% CI: 1.13–1.27) times the odds of screening compared with whites. This relative increase in screening appears to have been driven by higher uptake of flexible sigmoidoscopy in blacks compared with whites.

Results from this study are supported by previous studies investigating determinants of CRC screening. Previous studies have found that demographic characteristics such as income, race, education, and access to health insurance were significantly associated with receiving CRC screening within guidelines (19). Similar to results from the SCCS study, previous research has found that neighborhood deprivation, including higher rates of poverty, is associated with reduced uptake of CRC screenings (20). Somewhat unexpectedly, we found that full or part time work in the SCCS was associated with lower receipt of CRC screening. We hypothesize that unemployed individuals are more likely to be ill or disabled than those currently employed, and as such may have more frequent health-care visits than those who were employed, increasing their chance of receiving screening (21).

Our results also highlight the role of geographic disparities in CRC screening. We found that individuals residing in the Lower Mississippi Delta Region (a hotspot of CRC mortality) were significantly less likely to be within guidelines for CRC screening in comparison to individuals living outside of CRC mortality hotspot regions. These findings suggest not only that increased CRC mortality in the Lower Mississippi Delta region may be due to low utilization of CRC screenings but that the low uptake in this region is not simply a reflection of lower SES or other demographics, as other similarly low SES areas did not experience lower rates. Future research should target these underserved regions/populations with interventions designed to increase utilization of CRC screening.

Unpredictably, residence in two of the three hotspots was associated with increased odds of guideline-concordant screening. One possible explanation is that the reference group for screening rates within the SCCS (low-income, less educated, and underinsured) is quite different from the reference group used to identify the CRC mortality hotspots (the rest of the U.S.) (4). In addition, one must consider the continuum of care (22), follow-up, treatment, and access to care, which could contribute to increased CRC incidence and mortality in these regions. It is unknown if those who had an abnormal test result received follow-up care, nor do we know the quality of this follow-up care. Furthermore, the vast majority of health centers included in this study are federally qualified health centers or rural health clinics, located within designated health professional shortage areas (23) where such care may be particularly difficult to obtain.

### Strengths

The predominant strength of this study is the large sample size gathered from both study populations. In addition, both populations were recruited from regions of low SES in the U.S., further contributing to the body of knowledge surrounding uptake of cancer screenings within disadvantaged and underserved populations. Finally, the large geographic area from which these two samples were drawn may make these results generalizable to other Appalachian populations or regions of low SES.

### Limitations

We relied on self-report for assessment of screening within guidelines for both studies. For the OA data, medical records were reviewed to determine the accuracy of self-reported CRC screening within guidelines. Of the 2,482 individuals who self-reported CRC screening within guidelines, 615 patients either refused medical record access or there were issues with obtaining medical records from clinics. In total, medical record data were available for 1,867 individuals. Of these, 70% were accurate in their self-report of being within guidelines for CRC screening, as confirmed by medical record review. Medical records were not abstracted for individuals in the SCCS study, so it was impossible to confirm accuracy of self-reported screenings in the SCCS sample. Furthermore, the possibility of selection bias exists if the individuals who agreed to participate in the two studies are meaningfully different than those who refused participation—however, this is impossible to quantify.

Furthermore, limitations associated with differences between the study populations should be addressed. For one, each study population was different in terms of population size, characteristics, as well as geographic region assessed. Overall, the characteristics of participants in the SCCS study represented a lower SES than the OA participants, as a higher proportion of participants in the SCCS were uninsured, had low incomes, were unemployed or disabled and reported less than a high school education when compared with participants in the OA study. As such, participants were not representative of the general population. In addition, the inclusion of health centers from very rural areas in the SCCS can lead to some counties in the SCCS having very few numbers of participants, whereas other counties containing metropolitan areas or health centers with large patient populations may be more highly represented in this population.

## Conclusion

A parallel analysis demonstrated disparities in screening rates among residents of underserved communities, particularly those in geographic regions identified as having higher CRC mortality rates. This information can be used by health agencies and researchers to target intervention programs and efforts to these areas to increase CRC screening and decrease CRC mortality rates.

## Ethics Statement

This study was carried out in accordance with the recommendations of federal and state regulations governing the protections of human subjects. The protocol was approved by the Institutional Review Boards at Ohio State University, Vanderbilt University Medical Center and Meharry Medical College. All subjects gave written informed consent in accordance with the Declaration of Helsinki.

## Author Contributions

BB: methodology, writing-original draft preparation; writing-review and editing; AG: methodology, formal analysis, writing-original draft preparation; writing-review and editing; GY: methodology, formal analysis, writing-original draft preparation; RB: writing-review and editing; SR: conceptualization, writing-review and editing; WB and EP: conceptualization, investigation, writing-review and editing, supervision, funding acquisition.

## Conflict of Interest Statement

The authors declare that the research was conducted in the absence of any commercial or financial relationships that could be construed as a potential conflict of interest.
